# Predictive Metagenomic Analysis of Autoimmune Disease Identifies Robust Autoimmunity and Disease Specific Microbial Signatures

**DOI:** 10.3389/fmicb.2021.621310

**Published:** 2021-03-04

**Authors:** Angelina Volkova, Kelly V. Ruggles

**Affiliations:** ^1^Institute for Systems Genetics, New York University Grossman School of Medicine, New York, NY, United States; ^2^Division of Translational Medicine, Department of Medicine, New York University Grossman School of Medicine, New York, NY, United States

**Keywords:** microbiome, machine learning, autoimmune disease, metagenomics, metabolomics

## Abstract

Within the last decade, numerous studies have demonstrated changes in the gut microbiome associated with specific autoimmune diseases. Due to differences in study design, data quality control, analysis and statistical methods, many results of these studies are inconsistent and incomparable. To better understand the relationship between the intestinal microbiome and autoimmunity, we have completed a comprehensive re-analysis of 42 studies focusing on the gut microbiome in 12 autoimmune diseases to identify a microbial signature predictive of multiple sclerosis (MS), inflammatory bowel disease (IBD), rheumatoid arthritis (RA) and general autoimmune disease using both 16S rRNA sequencing data and shotgun metagenomics data. To do this, we used four machine learning algorithms, random forest, eXtreme Gradient Boosting (XGBoost), ridge regression, and support vector machine with radial kernel and recursive feature elimination to rank disease predictive taxa comparing disease vs. healthy participants and pairwise comparisons of each disease. Comparing the performance of these models, we found the two tree-based methods, XGBoost and random forest, most capable of handling sparse multidimensional data, to consistently produce the best results. Through this modeling, we identified a number of taxa consistently identified as dysregulated in a general autoimmune disease model including *Odoribacter, Lachnospiraceae Clostridium*, and *Mogibacteriaceae* implicating all as potential factors connecting the gut microbiome to autoimmune response. Further, we computed pairwise comparison models to identify disease specific taxa signatures highlighting a role for *Peptostreptococcaceae* and *Ruminococcaceae Gemmiger* in IBD and *Akkermansia, Butyricicoccus, and Mogibacteriaceae* in MS. We then connected a subset of these taxa with potential metabolic alterations based on metagenomic/metabolomic correlation analysis, identifying 215 metabolites associated with autoimmunity-predictive taxa.

## Introduction

The human intestine is colonized by millions of microbes, which have been shown to be involved in metabolism (Nicholson et al., 2012), immunity (Belkaid and Hand, 2014), and host physiology (Dominguez-Bello et al., 2019). This complex ecosystem has been extensively studied in the context of disease (Gilbert et al., 2016; Duvallet et al., 2017), diet (Carmody et al., 2015; Singh et al., 2017; Ruggles et al., 2018), and age (O’Toole and Jeffery, 2015) with the goal of determining how specific taxa and, more recently, the gene expression patterns of these taxa, impact human health. The relationship between the microbiome and the immune system has been of particular interest and specific bacteria have been shown to affect the function of both innate and adaptive immunity (Honda and Littman, 2016). Further, an increasing number of inflammatory and autoimmune disorders have been associated with microbial dysbiosis (Levy et al., 2017), though the precise mechanism for this relationship remains unclear.

Autoimmune diseases are multifactorial and chronic and the term covers nearly 100 distinct disorders (Wang et al., 2015). Although there appears to be some genetic component, studies in disease-discordant twins have found that concordance rates are incomplete and therefore environmental factors, including the gut microbiome, likely contribute to disease pathogenesis (Berer et al., 2017; Horta-Baas et al., 2017). Hundreds of studies have been carried out to better understand the connection between the microbiome and autoimmunity including studies specifically focused on inflammatory bowel disease (IBD), multiple sclerosis (MS), rheumatoid arthritis (RA), type 1 diabetes (T1D), and systemic lupus erythematosus (SLE). Despite the extensive study of the human gut microbiome in autoimmune disease, published results are inconsistent, which can be attributed to the differences in origin of samples (e.g., fecal or mucosal), sequencing platforms (Tremblay et al., 2015), sample sizes, therapies administered, patients’ age (O’Toole and Jeffery, 2015), geographical location (Yatsunenko et al., 2012), and methods of data analysis. Thus, the question of whether there are common microbial features characterizing general autoimmunity still remains.

Therefore, to better understand the role of specific taxa in autoimmunity, we have reprocessed and reanalyzed 42 16S and metagenomic studies focused on the gut microbiome and autoimmunity. To do this, we have taken advantage of several machine learning approaches to provide an alternative to the traditional diversity analysis (Knights et al., 2011; Statnikov et al., 2013; Mossotto et al., 2017). We specifically chose Random Forest (RF) (Breiman, 2001), eXtreme Gradient Boosting (XGBoost) (Chen and Guestrin, 2016), Support Vector Machine (Cortes and Vapnik, 1995) with Recursive Feature Elimination (Kohavi and John, 1997) (SVM RFE), and Ridge Regression (Hoerl and Kennard, 1970) algorithms since in addition to predicting a label they rank features according to how important the feature is for the label (disease) prediction. Random forest is a decision tree algorithm that has shown to be one of the most effective methods for classification of microbiome data, particularly 16S rRNA sequencing data (Statnikov et al., 2013). XGBoost, also a tree-based algorithm, has been recently shown to outperform other machine learning algorithms on a variety of biological datasets (Dimitrakopoulos et al., 2018; Ma et al., 2020). Further, we included ridge regression, another widely used algorithm that differs from these tree-based models in that it is a logistic regression algorithm with L2 regularization that still enables us to compare its feature ranking to other algorithms. Finally, we used SVM RFE since it is a powerful feature selection method that has been used in numerous biomedical applications (Hemphill et al., 2014). Moreover, many machine learning methods can handle sparse data with a large number of features, ranking them based on importance in their ability to distinguish between health and disease states (Kuhn and Johnson, 2013). These algorithms were used to identify microbial features predictive of general autoimmunity, as well as individual autoimmune diseases through the reanalysis of publicly available data on human gut microbiome in autoimmune diseases from the previous 10 years.

## Materials and Methods

### Data Acquisition

The NCBI PubMed database was searched for publications on April 1, 2020 related to the gut microbiome in autoimmune diseases from the last 10 years based on the following criteria: (1) the study was performed on human fecal samples; (2) the subjects in the studies were older than 2 years old; (3) the samples were sequenced with either 16S rRNA sequencing or shotgun metagenomics or both; (4) the raw data in FASTQ format were publicly available; (5) the provided metadata allowed us to distinguish between disease and healthy control samples, as well as between subjects who were explicitly treated in the study and untreated samples. We identified a total of 42 studies, 30 with 16S rRNA sequencing data, 9 with shotgun metagenomics and 3 studies with both types of data available. In order to balance the number of the subjects with autoimmune disease with the number of healthy controls, we added 2 additional 16S rRNA studies, from which we selected only the healthy controls. Also, we included healthy samples with both 16S rRNA and shotgun metagenomics data from Human Microbiome Project 1 (HMP1) (Supplementarxy Tables 1, 2).

### 16S rRNA Data Preprocessing

We employed QIIME2 (Bolyen et al., 2018) (v. 2018.11) to obtain the taxonomic abundances of the samples within each study, which were reprocessed independently and only the first time point was selected from each subject. Following data input, 454-based data underwent an error correcting step with *qiime dada2 denoise-pyro* (parameters: –p-trunc-len 0, –p-trim-left 20) command while the remaining samples were processed with either *qiime dada2 denoise-paired* (parameters: –p-trunc-len-f 0, –p-trunc-len-r 0, –p-trim-left-f 20, –p-trim-left-r 20) or *qiime dada2 denoise-single* (parameters: –p-trunc-len 0, –p-trim-left 2) commands depending on whether the reads were paired or single (Supplementary Table 2). The resulted sequence abundance tables were rarefied to the depth of 5,000. This depth was selected based on the alpha diversity curves of the studies, in which the plot reached a plateau. Further, we tried to account for 454-specific data since the sequencing depth of 454 samples was significantly lower than that of Illumina or Ion Torrent. As a result, the samples with sequencing depth less than 5,000 were excluded from the further analysis (Supplementary Figure 1). In the next step we assigned the taxonomy to the sequences by training a Naïve Bayes classifier on the entire 16S rRNA gene with *qiime feature-classifier fit-classifier-naive-bayes command* based on the Greengenes database (v 13_8) (DeSantis et al., 2006). Following taxonomy assignment, the taxonomic abundances tables were collapsed on both genus and species taxonomic levels. Further the resulting abundance tables from each study were merged together to create an “autoimmunity” data matrix and disease-specific matrices.

### Shotgun Metagenomics Preprocessing

KneadData (The Huttenhower Lab) was used to remove host sequences from reads by aligning the reads to the UCSC hg38 version of the human genome with the following Trimmomatic (Bolger et al., 2014) (v.0.36) parameters: ILLUMINACLIP:TruSeq3-SE:2:30:10 for single-end reads and ILLUMINACLIP:TruSeq3-PE.fa:2:30:10 for paired-end reads, LEADING:3,TRAILING:3, SLIDINGWINDOW:4:15, MINLEN:36. The resulted output was supplied to MetaPhlAn2 (Truong et al., 2015) to obtain relative taxonomic abundance, after which tables from individual studies were merged. One exception was the Cekanaviciute et al. (2017) study, for which only preprocessed tables were available, which were processed in the similar way (Supplementary Figure 2).

### Predictive Modeling

Caret package (Kuhn, 2008) in R was used to build the predictive models which were computed separately for each data type. For 16S rRNA we built 4 disease-specific models: autoimmune disease samples vs. healthy controls, IBD samples vs. healthy controls, MS samples vs. healthy controls and RA vs. healthy controls. We built those models on all samples that passed our inclusion criteria and on only adult (18 years and older) samples since children gut microbiomes have been shown to differ in diversity and composition compared with adults (Radjabzadeh et al., 2020). In addition, we built predictive models comparing IBD and MS, IBD and RA, and MS and RA. For MS and RA models only adult samples were used. All models were trained on both genus and species taxonomic levels. Since we identified only 13 studies with publicly available shotgun metagenomics data, we computed only 2 metagenomics models: all autoimmune disease samples vs. healthy controls model and IBD vs. healthy controls model. Also, since there were significantly more healthy samples than disease samples, when considering the individual disease models, we randomly selected the same number of healthy controls samples to match the number of available disease samples. We employed the same approach for the disease vs. disease models: the condition with the larger number of samples was randomly subsampled to match the number of samples in the condition with the smaller number of samples.

The data were split into training (90%) and test (10%) sets. The predictive models for each dataset were built with four algorithms: Random Forest (Breiman, 2001), XGBoost (Chen and Guestrin, 2016), Ridge Regression (Hoerl and Kennard, 1970) and SVM (Cortes and Vapnik, 1995) with radial kernel and RFE (Kohavi and John, 1997) with a step of 2. Those models were selected due to their ability to rank the features based on the importance for the label prediction. To reduce the computing time before the training step near-zero-variance features were identified and removed. In order to avoid overfitting and tune the parameters of the model, sevenfold-3-times cross-validation was employed. The final parameters, as well as the number of samples and features used for each model, are reported in Supplementary Table 3.

### Study Specific Models

In order to account for potential study-specific batch effects, we created “mock” models to predict the study a sample came from, regardless of disease status. To do this, we used a Random Forest model to predict study and then identified taxa features most predictive of each 16S (Supplementary Figures 3A,C) and metagenomics (Supplementary Figures 3A,C) study. We classified taxa as “predictive of study” if the feature was found to have a Gini importance greater than 68.5, a cutoff that was chosen because it filtered for taxa with the top 1% of importance values across model features tested (Menze et al., 2009).

### Feature Selection

Each of the selected algorithms ranked features based on their importance to the classification tasks we performed. Since the four algorithms employ different metrics for the feature ranking, we sorted the features in the descending order based on the importance in each algorithm. Further we selected the top 30 most important features for each of the models which were then collectively visualized using their mean rank average across models, in descending order.

### Metabolomic Analysis

For this purpose, we utilized the Inflammatory Bowel Disease Multiomics Database (IBDMDB) (Proctor et al., 2019), which is a part of iHMP (HMP2 in our dataset) that contains taxonomic, metagenomic, metatranscriptomic, metaproteomic, and metabolic data comparing the microbiome in IBD subjects and healthy controls. 382 samples had both metagenomic and metaproteomic data. For metagenomic data, we utilized the microbial abundance table that resulted from our analyses and for metabolomic data we downloaded the metabolites abundance table from the IBDMDB. Next we selected taxa that overlapped between at least one disease vs. disease models, that were identified on the genus level and were present in the IBDMDB dataset. This method provided 12 different genera, 2 of which were filtered out due to study-based predictive power (Supplementary Figure 3) and 4 of which were filtered based on the missingness cutoff (a taxon of interest has to be present in at least 10% of the HMP2 samples). In the next step we correlated the abundance of the remaining 6 genera in the IBDMDB with the metabolomics table from IBDMDB by using pairwise Spearman correlation with Benjamini-Hochberg correction for multiple comparisons and selected metabolites based on correlations with an adjusted *p*-value cutoff of 0.05.

All relevant code used for this project has been deposited here: https://github.com/avolkova1593/autoimmunity_paper.

## Results

### Autoimmunity-Associated Changes in Microbial Composition

**FIGURE 1:**
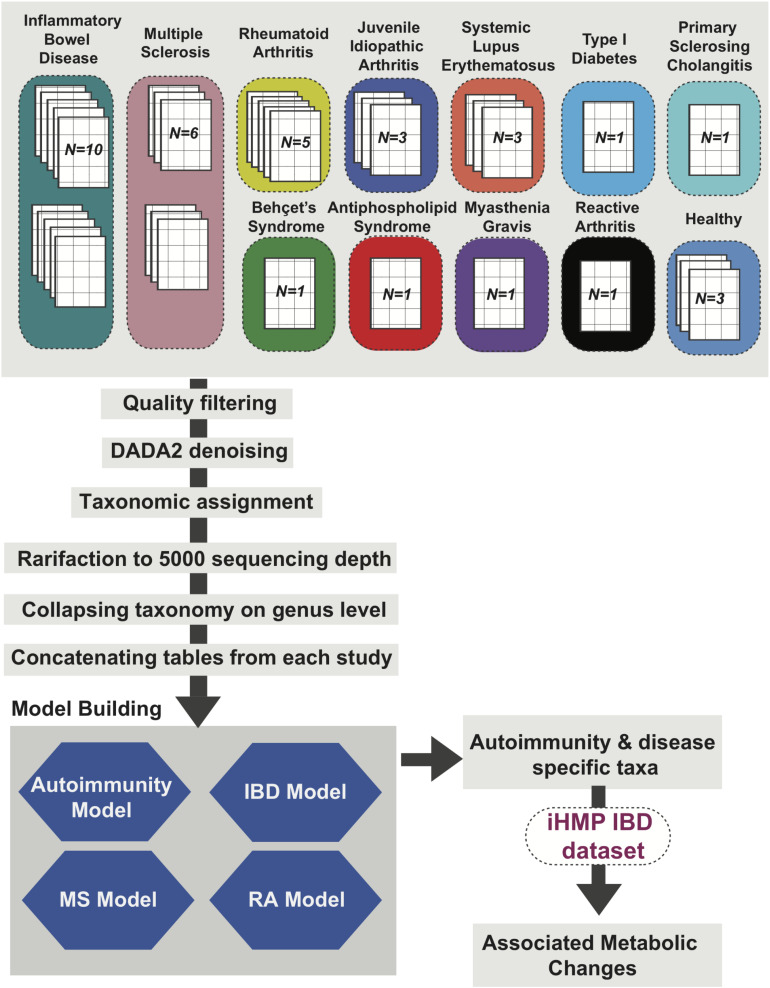
Autoimmunity Analysis Workflow. Thirty 16S rRNA sequencing datasets from studies focused on 12 different autoimmune diseases and three datasets representing healthy (non-autoimmune disease) cohorts were reprocessed using QIIME2 (Bolyen et al., 2018) with data2 denoising and rarified to 5,000 sequence depth. The output species and genus level relative abundance matrices were used to create four machine learning models for (1) general autoimmunity; (2) inflammatory bowel disease (IBD); (3) multiple sclerosis (MS); and (4) rheumatoid arthritis (RA). Top ranked features from these models were identified and metabolic changes associated with these taxa of interest were assessed using the IBDMDB dataset (Proctor et al., 2019).

We used a standardized meta-analysis approach to collect, reprocess and integrate available metagenomics data from case-control autoimmunity studies focusing on changes in the gut microbiome from human fecal samples. Using an expansive literature search we identified a total of 132 autoimmunity studies fulfilling our criteria (Supplementary Figure 1). Following filtering based on unique data, age (2 years or older), metadata and raw file availability and sequencing depth we were able to successfully download raw (FASTQ)16S rRNA and/or shotgun metagenomics data from 42 studies, 30 with 16S rRNA sequencing data (Hevia et al., 2014; Mejía-León et al., 2014; Stoll et al., 2014; Consolandi et al., 2015; Miyake et al., 2015; Chen et al., 2016a,b; Di Paola et al., 2016; Dunn et al., 2016; Jangi et al., 2016; Mar et al., 2016; Shaw et al., 2016; Tejesvi et al., 2016; Bajer et al., 2017; Halfvarson et al., 2017; Jacob et al., 2017; Pascal et al., 2017; Goyal et al., 2018; Luo et al., 2018; Manasson et al., 2018; Moris et al., 2018; Braun et al., 2019; Kozhieva et al., 2019; Lee et al., 2019; Li et al., 2019; Ruff et al., 2019; Sprockett et al., 2019; Sun et al., 2019; Zegarra-Ruiz et al., 2019; Choileáin et al., 2020) and 9 studies with shotgun metagenomics data (Lewis et al., 2015; Heintz-Buschart et al., 2016; Ananthakrishnan et al., 2017; Hall et al., 2017; Wen et al., 2017; Ye et al., 2018; Proctor et al., 2019; Ventura et al., 2019; Zhou et al., 2020), and 3 studies with both (Scher et al., 2013; Cekanaviciute et al., 2017; Connors et al., 2020; Supplementary Table 1 and Supplementary Figure 1). These included studies on Inflammatory Bowel Disease (IBD, *N* = *14*), Multiple Sclerosis (MS, *N* = *7*), Rheumatoid Arthritis (RA, *N* = *5*), Juvenile Idiopathic Arthritis (JIA, *N* = *3*), Systemic Lupus Erythematosus (SLE, *N* = *3*), Type 1 Diabetes (T1D, *N* = *2*), Behcet’s Syndrome (BS, *N* = *2*), Ankylosing Spondylitis (AS, *N* = *2*), Antiphospholipid Syndrome (APS, *N* = *1*), Primary Sclerosing Cholangitis (PSC, *N* = *1*), Myasthenia Gravis (MG, *N* = 1) and Reactive Arthritis (ReA, *N* = *1*) (Figure 1 showing 16S study Ns, Supplementary Figure 2 showing metagenomic study Ns). Three additional studies with healthy subjects were included to balance the disease and non-diseased cohorts (Supplementary Tables 1, 2).

Initially, 16S rRNA data was reprocessed using a standard analysis pipeline, which included filtering and taxonomic assignment. Each study was reprocessed individually and final taxonomic abundance tables were then concatenated to a build a final autoimmunity matrix. Disease specific datasets were also created through combining reprocessed data tables for each individual disease type. Each table was then used to build predictive models of general autoimmunity as well as disease-specific models (Figure 1) with the primary goal of identifying the most important features (taxa) involved in autoimmunity across and within disease types. Metagenomics data was also reprocessed using a separate analysis pipeline, providing taxonomic abundance tables (Supplementary Figure 2).

**FIGURE 2:**
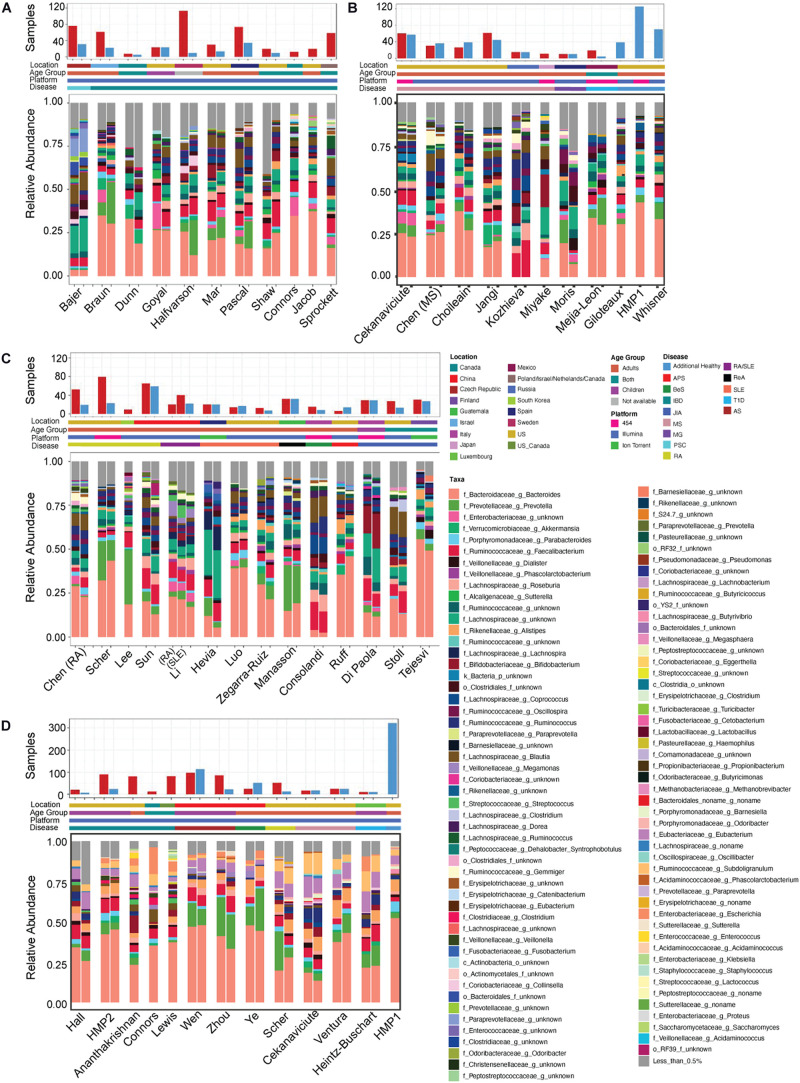
Study overview. Overview of **(A–C)** 16S rRNA sequencing and **(D)** shotgun metagenomics studies included in our analysis. Includes the number of healthy (blue bars) and disease (red bars) samples in each, geographic location, age group and disease studied. Also includes the average relative abundance of taxa at the genus level for the healthy and diseased subjects following re-processing. Inflammatory bowel disease (IBD), multiple sclerosis (MS), rheumatoid arthritis (RA), juvenile idiopathic arthritis (JIA), systemic lupus erythematosus (SLE), type 1 diabetes (T1D), primary sclerosing cholangitis (PSC), Behcet’s syndrome (BeS), ankylosing spondylitis (AS), antiphospholipid syndrome (APS), myasthenia gravis (MG), and reactive arthritis (ReA).

Following quality control (QC) and filtering, 33 studies containing 16S rRNA (Scher et al., 2013; Hevia et al., 2014; Mejía-León et al., 2014; Stoll et al., 2014; Consolandi et al., 2015; Miyake et al., 2015; Chen et al., 2016a,b; Di Paola et al., 2016; Dunn et al., 2016; Jangi et al., 2016; Mar et al., 2016; Shaw et al., 2016; Tejesvi et al., 2016; Bajer et al., 2017; Cekanaviciute et al., 2017; Halfvarson et al., 2017; Jacob et al., 2017; Pascal et al., 2017; Goyal et al., 2018; Luo et al., 2018; Manasson et al., 2018; Moris et al., 2018; Braun et al., 2019; Kozhieva et al., 2019; Lee et al., 2019; Li et al., 2019; Ruff et al., 2019; Sprockett et al., 2019; Sun et al., 2019; Zegarra-Ruiz et al., 2019; Choileáin et al., 2020; Connors et al., 2020) and 12 studies containing metagenomics (Scher et al., 2013; Lewis et al., 2015; Heintz-Buschart et al., 2016; Ananthakrishnan et al., 2017; Cekanaviciute et al., 2017; Hall et al., 2017; Wen et al., 2017; Ye et al., 2018; Proctor et al., 2019; Ventura et al., 2019; Connors et al., 2020; Zhou et al., 2020) data remained for downstream analysis (Figure 1 and Supplementary Figure 2). Notably, 10 out of the 33 16S rRNA and 5 of the 12 metagenomics data sets investigated the role of the human gut microbiome in IBD, due in part to its relatively high prevalence in 1.3% of US adults (CDC, 2019). However, we were also able to acquire data from studies of more rare autoimmune diseases including Behçet’s Syndrome, which results from inflammation of the blood vessels (Consolandi et al., 2015), Myasthenia Gravis, a neuromuscular disorder, and Reactive Arthritis. A portion of these studies contained significantly more disease samples than the healthy samples, with Halfvarson et al. (2017) having 10 times more samples from individuals with autoimmune disease than from healthy controls, and with 6 other studies (Lewis et al., 2015; Ananthakrishnan et al., 2017; Jacob et al., 2017; Lee et al., 2019; Sprockett et al., 2019; Connors et al., 2020) containing only disease samples (Figure 2). For this reason, we included healthy samples from three additional studies which investigated non-autoimmune diseases (Huttenhower et al., 2012; Giloteaux et al., 2016; Whisner et al., 2018), which after QC and preprocessing resulted in additional 232 16S and 322 shotgun metagenomics samples.

While combining these diverse datasets there were several study-specific characteristics known to impact microbial identification that we paid specific attention to, such as geography, age, sequencing platform and 16S rRNA primers. A majority of the studies were based on populations from North America, Europe and Asia, however Manasson et al. (2018) investigated the gut microbiome of spondyloarthritis in Guatemalan patients, Mejía-León et al. (2014) looked at Type 1 Diabetes in Mexico, while Sprockett et al. (2019) had participants from four different countries, Poland, Israel, Netherlands and Canada. Further, there was a large range in age across studies, with participants being from 2 to 76 years old. Studies focusing on newborn children (less than 2 years of age) were not included since it has been well established that the microbial diversity in the first few years of life is significantly lower when compared with adults (Korpela and de Vos, 2018). We also controlled for age by building separate models for adults only (18 years or older) in addition to models including all participants in datasets where children were included (general autoimmunity and IBD). DNA was sequenced with one of three sequencing platforms, 454 pyrosequencing, Ion Torrent, or Illumina instruments with both paired and single reads techniques. Description of the characteristics for each study can be found in Supplementary Table 2 and Figure 2. To assess potential batch effects, we employed a Principal Coordinate Analysis (PCoA) (Gower, 1966) based on the Bray-Curtis distance (Beals, 1984) and investigated disease and non-disease based differences. All variables are shown by PCoA and, as expected, were found to have significant differences based on an Adonis test (*p* < 0.001) (Supplementary Figures 4, 5). To combat this, we completed study-based analysis to identify study-specific vs. disease-specific features as part of our downstream analysis (Supplementary Figure 3).

We first examined the taxonomic composition on the genus level of the healthy and diseased samples in each study to verify expected changes based on previously published results. We were able to recapitulate major findings from all studies. For example, we identified disease-specific alterations in multiple studies in *Akkermansia* (Jangi et al., 2016; Kump et al., 2018), *Bacteroides* (Hevia et al., 2014; Dunn et al., 2016; Zhou et al., 2020), *Blautia* (Luo et al., 2018; Manasson et al., 2018), *Clostridiaceae* (Di Paola et al., 2016), *Faecalibacterium* (Stoll et al., 2014; Chen et al., 2016b), *Lachnospira* (Stoll et al., 2014; Mar et al., 2016; Halfvarson et al., 2017), *Parabacteroides* (Cekanaviciute et al., 2017), *Prevotella* (Mejía-León et al., 2014; Mar et al., 2016; Wen et al., 2017; Manasson et al., 2018; Zhou et al., 2020), *Ruminococcacaea* (Dunn et al., 2016; Mar et al., 2016; Halfvarson et al., 2017; Kump et al., 2018; Manasson et al., 2018), and *Streptococcus* (Chen et al., 2016b; Figure 2). Interestingly, these previously published results, and our reanalyzed results, varied in the directionality of the change for many of these taxa, with disease specific overabundance occurring in a subset of studies and a reduction in other. These inconsistencies further highlight the need for standardized reanalysis and integration of these valuable datasets to better understand the potential impact of microbial changes in autoimmune disease.

The taxonomic composition of healthy individuals showed clear differences, which can be attributed to several factors. First, it is well established that microbial composition differs by age and geography (Yatsunenko et al., 2012). Secondly, it is not guaranteed that the “healthy” recruits included in these studies did not suffer from another pathology impacting the gut microbiome. In most studies, researchers only ensured that healthy controls had not been diagnosed with an autoimmune disease of interest and had not taken antibiotics at least during the sample collection. Thirdly, as these studies were sequenced on different platforms and with differing 16S rRNA hypervariable regions during PCR amplification, we expect a level of variability in the identified taxa even across controls (Fredriksson et al., 2013).

### Predictive Modeling of Autoimmunity

In order to identify which taxa are most important for distinguishing between healthy controls and subjects with autoimmune disease we built four independent machine learning disease models on 16S rRNA data: (1) IBD specific; (2) MS specific; (3) RA specific; and (4) general autoimmunity; which included samples from all the autoimmune diseases available (Figure 1). Genus level taxonomic abundances were used for the final predictive modeling analyses. Four independent algorithms were used to capitalize on the strengths and limitations of each: Random Forest (RF) (Breiman, 2001), eXtreme Gradient Boosting (XGBoost) (Chen and Guestrin, 2016), Support Vector Machine (Cortes and Vapnik, 1995) with Recursive Feature Elimination (Kohavi and John, 1997) (SVM RFE), and Ridge Regression (Hoerl and Kennard, 1970). For both the general autoimmunity and IBD model, an “Adult only” model was also created, removing all participants younger than 18 years old, to control for known age-specific differences in microbial composition. MS and RA models included only adults. Application of four independent algorithms capable of feature ranking to the same data provided an advantage in robustly identifying the most important features predictive of autoimmunity by multiple models, providing an additional level of confidence. Models were run at both the genus and species level.

**FIGURE 3:**
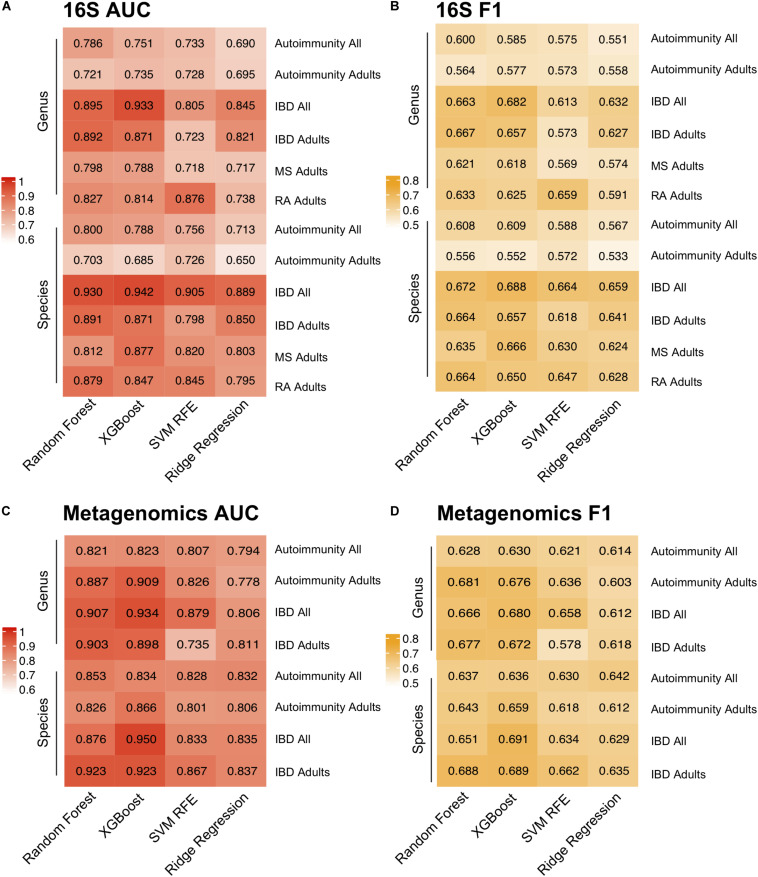
Predictive Modeling of Autoimmune Disease. Area under the curve (AUC) **(A,C)** and F1-scores **(B,D)** for models predicting any autoimmune disease, inflammatory bowel disease (IBD), multiple sclerosis (MS) and rheumatoid arthritis (RA) for four machine learning models, random forest, XGBoost, support vector machine with recursive feature elimination (SVM RFE) and ridge regression and at the genus and species level. Adult-only (18 years and older) and All (adult + children) models are included for general autoimmunity and IBD studies.

Model performance was evaluated using both Area Under the receiver operating characteristics Curve (AUC) and macro F1 score, which reports the balance between the precision and the recall. Notably, we incorporated near-zero-variance feature removal to reduce both computational load and to consider only features with reasonable variation between the samples, as those with little variation likely would not impact disease state. Among the four algorithms for the autoimmunity model, the best performance was achieved by Random Forest with an AUC of 0.8 using the species level data. The superior performance by this algorithm was not unexpected, as Random Forest has been previously shown to perform well on microbial data (Statnikov et al., 2013). Random Forest was also the best predictor for the species-level RA model, with an AUC of 0.879. XGBoost produced the best AUC at the species level for the IBD and MS disease prediction of 0.942 and 0.877, respectively (Figure 3). In general, model performance was similar at the species and genus level, with slightly higher AUCs occurring in the species models. In addition, we applied the same predictive modeling strategy to shotgun metagenomics data. Due to data availability, we built only general autoimmunity and IBD models, with highest AUCs for “Adult only” models reaching 0.866 for the general autoimmunity model and 0.923 for the IBD model using the species level data.

Overall, the most stable AUCs across the three algorithms was reached on the IBD data set, likely due to the considerably higher number of IBD samples compared with other autoimmune diseases. Notably, we were able to predict autoimmunity based on only microbial composition of the samples, which suggests that there exists a common gut microbiome signature present that may be relevant to all autoimmune diseases. In order to determine whether our AUCs could be predicted by chance, we assigned the labels to the samples at random, and computed our models again. The models trained with the random label assignment produced the AUCs of ∼0.5 (Supplementary Figure 6), which is indicative of a true difference between the healthy controls and autoimmune disease subjects based on the gut microbial composition.

### Most Predictive Model Features

**FIGURE 4:**
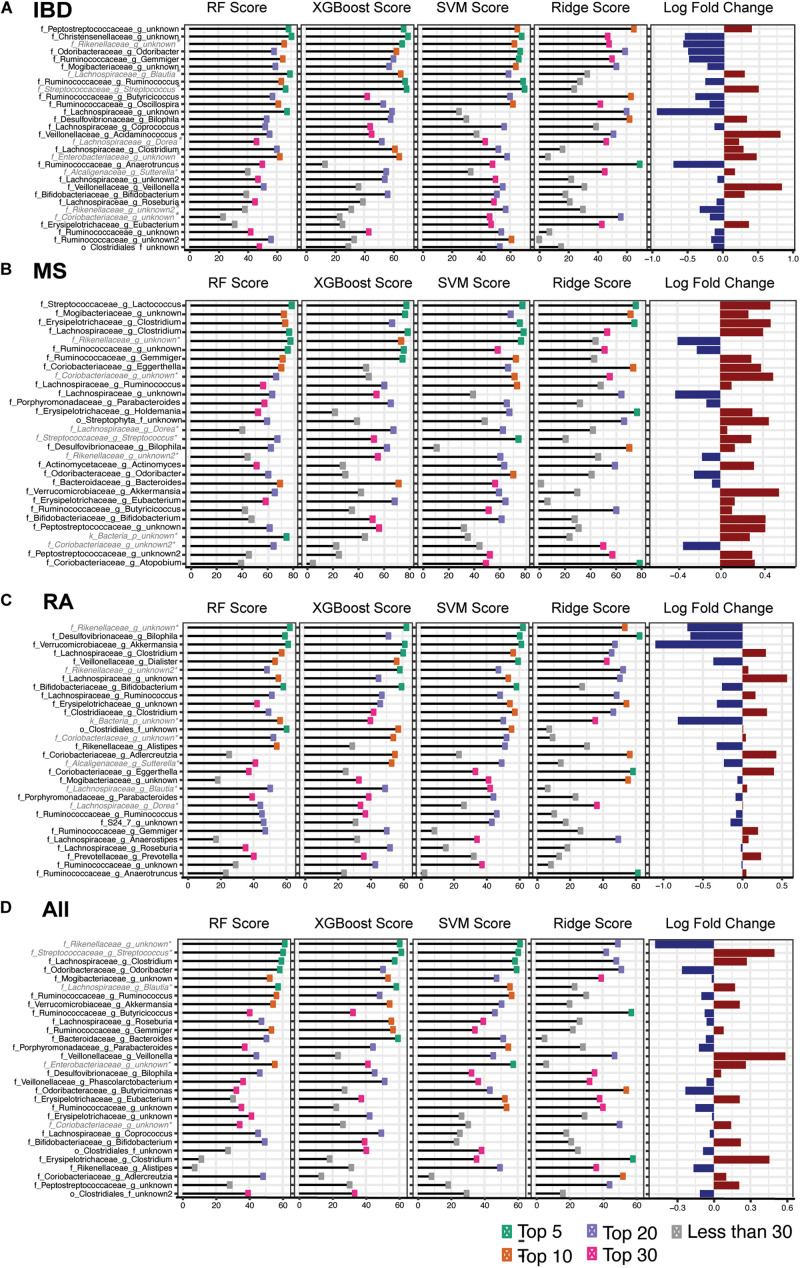
Taxa Predictive of Disease Top 30 taxa across four predictive models (adults only), random forest (RF), XGBoost, and support vector machines (SVM), ridge regression for **(A)** inflammatory bowel disease **(B)** multiple sclerosis, **(C)** rheumatoid arthritis and **(D)** general autoimmunity. Features ranked by mean rank across the four models in descending order and color indicates the rank of each taxa in each model. Log fold change of disease vs. healthy for each taxon. *Indicates that the taxa highlighted was identified as being highly study specific (Supplementary Figure 3).

Since all four of our models employed feature ranking we were able to identify which features were most important for predicting the three distinct autoimmune diseases as well as general autoimmunity. From this, we identified features that were ranked highly by all four algorithms. The top 30 features were selected based on a combined feature score of ranked taxa across all 4 models for each disease (Figure 4 and Supplementary Figure 7). This combined feature ranking approach allowed us to focus specifically on the most confident set of features in our dataset that were commonly identified by all four classification approaches.

In order to account for potential batch affects occurring due to study population differences (Supplementary Figures 4, 5), we created “mock” models to predict the study a sample came from, regardless of disease status. This allowed us to identify taxa that were able to specifically identify a study population rather than the disease. These models identified *Coriobacteriaceae*, *Bacteroidales, Rikenellaceae, Streptococcaceae Streptococcus, Lachnospiraceae Blautia, Lachnospiraceae Dorea, Alcaligenaceae Sutterella*, and *Enterobacteriaceae* as able to predict study regardless of disease or healthy status in at least one 16S rRNA study and *Ruminococcaceae Faecalibacterium, Desulfovibrionaceae Bilophila*, and *Enterobacteriaceae Escherichia* in the metagenomics studies (Supplementary Figure 3). This allowed us to identify taxa that are likely tied to the study population, sequencing platform or experimental method, rather than disease status.

The most predictive features identified by our IBD model were reduced levels of *Christensenellaceae, Odoribacter*, and *Gemmiger* and increased abundance of *Peptostreptococcaceae* (Figure 4A). MS predictive features included increases in *Lactococcus*, *Mogibacteriaceae*, *Erysipelotrichaceae Clostridium*, and *Lachnospiraceae Clostridium* and reduced levels *Ruminococcaceae* (Figure 4B). Further, the RA model identified reduced abundance of *Desulfovibrionaceae Bilophila*, *Akkermansia*, and *Veillonellaceae Dialister* and increased levels of *Lachnospiraceae Clostridium* as most predictive of disease state (Figure 4C). Lastly, for our comprehensive autoimmunity analysis, we identified *Odoribacter* and *Mogibacteriaceae* as the most important features with reduced abundance in autoimmune disease samples compared with healthy controls and *Clostridium* having increased expression in diseased participants (Figure 4D). Although *Rikenellaceae* was repeatedly identified by all disease models, our study-specific models also identified this genus as being highly study specific for one of the additional healthy control cohorts (HMP1) and therefore we did not consider it in our downstream biological interpretation (Supplementary Figure 3).

**FIGURE 5:**
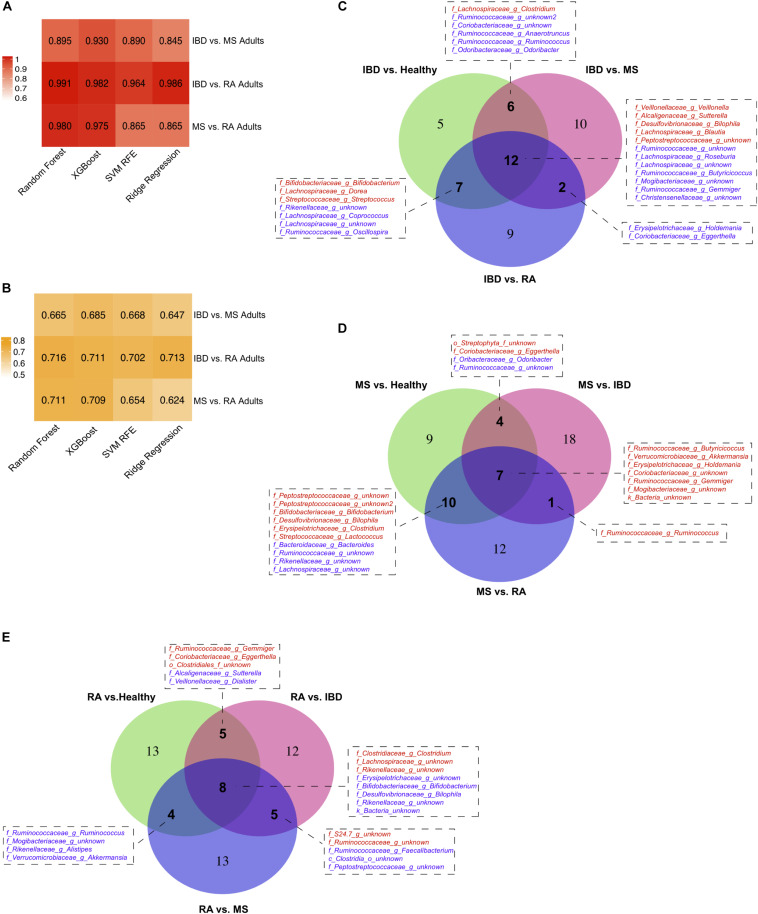
Disease vs. disease comparison models. Model **(A)** AUCs and **(B)** F1 scores when predicting diseased samples when compared against other disease. Taxa consistently identified in multiple comparison models for **(C)** inflammatory bowel disease (IBD), **(D)** multiple sclerosis (MS), and **(E)** rheumatoid arthritis (RA).

By also comparing our three disease types (IBD, MS, and RA) to each other we were able to further refine our disease specific predictive taxa from our heterogeneous dataset. To do this, we again used predictive modeling (Random Forest) to compare each disease to each other, identifying a new set of predictive taxa, and overlapped these with those identified in the original model created based on healthy controls. The model performance (AUC, F1 score) and overlap of the thirty most predictive taxa from each model is shown in Figure 5. This analysis provided us with a list of taxa able to distinguish each disease not only from healthy controls, but from other autoimmune diseases. In IBD, 12 features were identified in all three comparisons, including increased *Peptostreptococcaceae*, and decreased levels of *Mogibacteriaceae* and *Gemmiger* (Figure 5C). Increased *Butyricicoccus, Akkermansia, and Holdemania* were three of the seven taxa consistently predicted in our MS models (Figure 5D) and increased *Clostridiaceae Clostridium* and *Lachnospiraceae* and reduced *Erysipelotrichaceae* were three of the eight identified in all RA models (Figure 5E).

To validate these findings, we also applied the same machine learning approach to shotgun metagenomics data from 13 studies (Huttenhower et al., 2012; Scher et al., 2013; Lewis et al., 2015; Heintz-Buschart et al., 2016; Ananthakrishnan et al., 2017; Cekanaviciute et al., 2017; Hall et al., 2017; Wen et al., 2017; Ye et al., 2018; Proctor et al., 2019; Ventura et al., 2019; Connors et al., 2020; Zhou et al., 2020; Supplementary Figure 2). Six of the top 15 features most predictive features overlapped in both the 16S autoimmunity (Figure 4D) and metagenomics autoimmunity adult models (Supplementary Figure 7E), including *Clostridium, Odoribacter*, and *Parabacteroides*. Similarly, both 16S (Figure 4A) and metagenomics (Supplementary Figure 7F) IBD models had 3 overlapping top features including *Odoribacter* and *Ruminococcus*.

### Correlations Between Highly Ranked Taxa and Metabolism in IBD

**FIGURE 6:**
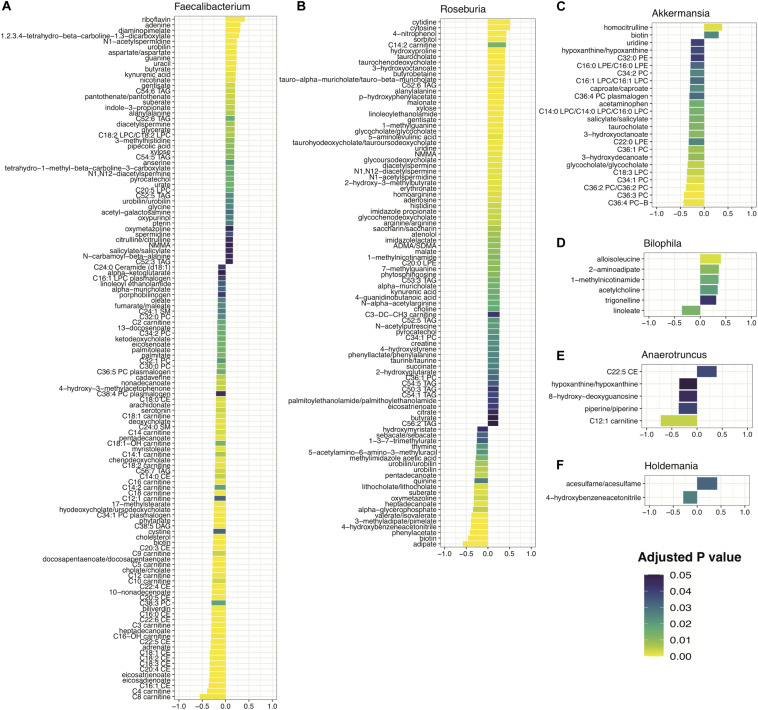
Metabolites significantly correlated with disease-predictive taxa: **(A)** Faecalibacterium, **(B)** Roseburia, **(C)** Akkermansia, **(D)** Bilophila, **(E)** Anaerotruncus, and **(F)** Holdemania. Spearman correlation coefficient scores plotted and shaded by adjusted *p*-value for 6 taxa found to be predictive of IBD, MS, and RA based on the multiple disease model comparisons.

To better understand the potential downstream effects of altered abundance levels of these taxa, we used the Inflammatory Bowel Disease Multiomics Database (IBDMDB) metabolomic dataset to identify metabolites which are significantly correlated with our taxa of interest. For this purpose, we chose features that overlapped in at least two of the three disease vs., disease models that identified on the genus level (25 taxa total, Figures 5C–E) and which were present in the IBDMDB shotgun metagenomics dataset. This resulted in a total of 12 genera in common between our dataset and IBDMDB cohort (Figure 6 and Supplementary Figure 8). A total of 6 taxa were excluded from the further analysis due to the following reasons. Two of these taxa (*Dorea, Sutterella*) were excluded from this analysis as they were also flagged as being consistently able to predict 16S study regardless of disease or healthy status of the samples (Supplementary Figure 3), and another four taxa *(Butyricicoccus, Eggerthella, Lactococcus, Odoribacter)* were filtered based on missingness (>90% missing) in the metagenomics data set.

Investigating correlations between the abundance of the remaining 6 genera with metabolites within the IBDMDB, we identified 215 metabolites that significantly correlated with at least one taxon at an adjusted *p* < 0.05. One of the 6 genera assessed, *Roseburia, was* found to be reduced in IBD (Figure 5C). It had the highest correlations occurring in a number of bile acids (e.g., taurocholate, taurochenodeoxycholate, glycocholate), in addition to several triacylglycerols (TAGs) and fatty acids (Figure 6B). Specifically, the short chain fatty acid (SCFA) butyrate was found to be positively associated with *Roseburia* abundance, consistent with known butyrate production in the *Roseburia* genus (Tamanai-Shacoori et al., 2017). Bacterial fermentation of carbohydrates in the gut are known to produce SCFAs and butyrate, in particular, has been established as playing a critical role in host metabolism (Kasubuchi et al., 2015) and intestinal anti-inflammatory action through NF-kB inhibition in colonic epithelial cells (Canani et al., 2011) and regulatory T cell (Treg) and T helper cell 17 (Th17) response (Arpaia et al., 2013; Smith et al., 2013). Further, *Roseburia* has a known anti-inflammatory role in the intestine (Lin and Zhang, 2017) and it’s positive association with butyrate is consistent with a deficiency in this genus being highly predictive of IBD (Figure 5C).

In models of RA, reduced abundance of *Faecalibacterium* was found to be predictive of disease (Figure 5E) and this bacterium was found to be significantly positively correlated with a number of metabolites including the vitamin B metabolites riboflavin, nicotinate and pantothenate; nucleotides adenine, guanine and uracil; and the SCFA butyrate. As a butyrate producer, *Faecalibacterium* is generally considered beneficial (Sokol et al., 2008), a point that is further highlighted by its positive correlation with B vitamins which have been shown to play important roles in immune function and both dietary and gut-derived vitamin B help to modulate immune homeostasis (Suzuki and Kunisawa, 2015; Hosomi and Kunisawa, 2017). *Faecalibacterium* was also found to be negatively associated with a number of acylcarnitines and cholesterol esters (Figure 6A).

Increased abundance of *Akkermansia* and *Holdemania* were found to be predictive of MS (Figure 5D). *Akkermansia* showed negative associations with the bile acid components taurocholate, bile acid glycocholate and fatty acid anions 3-hydroxyoctanoate and caproate (Figure 6C). The identification of bile acids associated with a number of our taxa is consistent with several studies showing an integral role of the gut bile acid pool as a modulator of host immune response and inflammation (Hang et al., 2019; Song et al., 2020). *Holdemania* had increased levels of the artificial sweetener, acesulfame, and the benzyl cyanide, hydroxybenzene acetonitrile (Figure 6F). Lastly, *Bilophila*, was found to be increased in RA (Figure 5E) and positively associated with the branched chain amino acid alloisoleucine and the lysine metabolite, 2-aminoadipate (Figure 6D).

## Discussion

In this analysis, we used data from 42 studies investigating the role of the human gut microbiome in autoimmune disease, assessing both general autoimmunity, and specific diseases. Since it is not always possible to find consistent differences using traditional meta-analysis methods, we applied classification algorithms to predict whether a sample comes from a healthy control or an autoimmune disease sample across multiple studies. We specifically used random forest, XGBoost, ridge regression and SVM RFE as these algorithms are capable not only predicting the disease status of the samples, but also ranking the features based on how important they are for the prediction.

Random forest has a long history in microbiome studies and has proven to be a robust algorithm that performs well on sparse unbalanced datasets. XGBoost is quickly gaining popularity and uses both types of regularization: L1 and L2 which prevents model from overfitting and it has in-built capability of handling sparse data and missing values. While random forest employs a bagging strategy, where each tree is provided with a full set of features and a sample of the data with replacement, XGBoost uses a boosting strategy, which is based on sequential training of shallow trees where each tree tries to correct the errors by the previous trees. Interestingly, both algorithms showed similar performance on our datasets. Further, we applied SVM RFE with a radial kernel, an algorithm that defines a non-linear hyperplane that maximizes the boundary between the two classes. In addition, SVM RFE utilizes recursive feature elimination, which is a wrapper algorithm that starts by training the model with all the features where the least important feature is eliminated, and it repeats this process until the best performance is reached. Due to the need to train the model repeatedly, SVM RFE requires significantly more training time than a regular SVM. For comparison we also used ridge regression, which is logistic regression with L2 regularization. Since it is a simpler model that captures only linear relations, which might be not sufficient enough to capture connections in the microbiome community, it did not perform as well as the other three models. A recent paper by Topçuoğlu et al. (2020) evaluating the application of 7 diverse machine learning models to microbiome data found similar results to ours, with tree-based models performing best but with logistic regression with L2 regularization closely following.

Connections between the gut microbiome and general autoimmunity have been made by studies investigating the role of human leukocyte antigen (HLA) gene polymorphisms in autoimmunity risk in a number of diseases including type 1 diabetes (Jerram and Leslie, 2017), spondyloarthritis (Kopplin et al., 2016), Behcet’s disease (Ohno et al., 1982), and Celiac disease (Karell et al., 2003) explained through the impact of HLA on the amino acid sequence in class II major histocompatibility complex (MHC). It has been hypothesized that these polymorphisms may be involved in immune response in the gut and could be a link between autoimmune disease and the microbiome composition (Russell et al., 2019). Interestingly, one of the top features identified by our IBD model, *Peptostreptococcaceae* (Figure 4), was also identified as being associated with HLA risk alleles in a T1D risk study. This taxa was found to be significantly associated with a lower HLA genetic risk of autoimmunity, identifying it as a potential environmental trigger for autoimmune disease and warranting further study in IBD genetic risk based on our results (Russell et al., 2019). We also identified a number of additional genera that were consistently predictive of disease. For example, reduced levels of *Lachnospiraceae Clostridium* and *Mogibacteriaceae* were identified as a top feature in all four of our disease models and serve as possible factors further connecting the gut microbiome and autoimmunity (Figure 4).

In addition to identifying taxa predictive of general autoimmunity we were able to identify a number of novel taxa specific to IBD, MS, and RA. Although several of these taxa have been previously associated with these diseases, conflicting and inconsistent results have been common. To try to circumvent these limitations, we have reanalyzed a large number of available gut microbiome studies to provide a broad perspective on the connection between the microbiome and specific disease. Our analysis has recapitulated several recent articles connecting the microbiome with autoimmunity and has also identified a number of novel taxa that may be related to these pathologies. For example, we found a depletion in *Roseburia*, *Ruminococcaceae* in IBD compared with controls, consistent with other studies of IBD (Duvallet et al., 2017) and identified *Akkermansia* as a consistently predictive taxa for MS, an organism which has been shown to interact with spore-forming bacteria to worsen the impact of MS-associated microbiota (Cekanaviciute et al., 2018).

Further, 6 of the taxa we identified as being predictive of autoimmune disease were correlated with metabolites that have been potentially involved with autoimmunity and inflammation. Recent publications have identified a number of bile acids (Hang et al., 2019; Song et al., 2020), triacylglycerols (Franzosa et al., 2019), vitamin B (Salem and Wadie, 2017; Lloyd-Price et al., 2019), and acylcarnitine (Lloyd-Price et al., 2019) metabolites involved immune response and the microbiome, many of which we also found to be significantly associated with our most predictive taxa. Histamine, along with taurine and spermine which were also highlighted by our analysis, have been found to help shape the host-microbiome relationship through the regulation of the NLRP6 inflammasome signaling (Levy et al., 2015). Further, we identified an association between IBD and RA predictive taxa, *Roseburia and Faecalibacteria*, with the SCFA butyrate, which among other SCFAs has been shown to inhibit histone deacetylases (HDACs) and inhibit immune response through Treg regulation and as ligands for G-protein coupled receptors with downstream anti-inflammatory effects (Smith et al., 2013; Rooks and Garrett, 2016; Haase et al., 2018). The association identified between metabolites and taxa could be either due to the impact of that metabolite on the growth of the taxa, the metabolite being a produced by said taxa, or the metabolite negatively associating growth of an inhibitory species, and thus must be followed up by a more targeted approach to understand the precise biological mechanism.

Duvallet et al. (2017), completed a similar meta-analysis study in 2017 looking across 10 disease types (arthritis, autism spectrum disorder, Crohn’s disease, *Clostridium difficile* infection, liver cirrhosis, colorectal cancer, enteric diarrheal disease, HIV infection, liver diseases, minimal hepatic encephalopathy, non-alcoholic steatohepatitis, obesity, Parkinson’s disease, psoriatic arthritis, rheumatoid arthritis, type I diabetes, and ulcerative colitis) to identify disease-specific and shared taxa. They too, identified a number of genera associated with more than one disease, including *Lachnospiraceae and Ruminococcaceae* families and several members of the *Lactobacillales* order and showed the strengths of cross disease comparison using publicly available data. Studies delving into specific disease subcategories, such as this study focused on autoimmune disease, build upon their original study. Further, our reanalysis focused more acutely on investigation of inter-study batch effects and methods of reducing the impact of these on downstream analysis. Since our dataset is immensely heterogenous, we had to tackling this issue creatively. Before using machine learning we used a percentile normalization approach implemented in QIIME 2 which was unable to address the batch effects in his dataset and therefore was not used in our downstream analyses and disease vs. healthy or disease vs. disease models.

We understand there are several limitations of this study. Firstly, the sample size is relatively small for machine learning reducing model reliability. As additional data is generated on larger cohorts from different ages and different cultural backgrounds we can continue to develop and run similar models to further elucidate how gut microbiome promotes autoimmune diseases. Additionally, the differences in sequencing platform, geography and subject characteristics provide confounders that are difficult to remove from the dataset *post hoc*. Cautious evaluation of taxa identified by our methods in addition to the use of control models testing the ability to predict by study rather than disease were used to combat this issue, however we are aware that these confounders remain. Future analysis further evaluating how each of these study design techniques and participant make-up effects the results of a microbiome study would be of great benefit to the community.

## Data Availability Statement

The original contributions presented in the study are included in the article/Supplementary Material, further inquiries can be directed to the corresponding author/s.

## Author Contributions

AV and KR contributed to the conception and design of the study. AV performed the data processing and predictive modeling and wrote the first draft of the manuscript. KR revised and completed the final draft of the manuscript. Both authors contributed to manuscript editing and approved the submitted version.

## Conflict of Interest

The authors declare that the research was conducted in the absence of any commercial or financial relationships that could be construed as a potential conflict of interest.

## Supplementary Material

Supplementary Figure 1 Study collection and filtering.

Supplementary Figure 2 Metagenomic analysis workflow.

Supplementary Figure 3 Models and features predictive of study. AUCs of random forest model prediction of study and taxa features most predictive of study in those models in 16S **(A,C)** and metagenomics **(B,D)** studies.

Supplementary Figure 4 PCoA diagrams and statistical differences across 16S datasets showing sample similarity by **(A)** health status, **(B)**16S rRNA region sequenced, **(C)** sequence platform, **(D)** disease type, **(E)** age group, **(F)** exact age, **(G)** country, **(H)** study, **(I)** antibiotics consumption, and **(J)** DNA extraction kit. All PCoAs have an Adonis *p* < 0.001.

Supplementary Figure 5 PCoA diagrams and statistical differences across metagenomics datasets showing sample similarity by **(A)** health status, **(B)** age group, **(C)** disease type, **(D)** exact age, **(E)** country, **(F)** antibiotics consumption, **(G)** study, and **(H)** DNA extraction kit. All PCoAs have an Adonis *p* < 0.001.

Supplementary Figure 6 AUCs for models trained with random label assignment for **(A)** 16S and **(B)** metagenomics studies.

Supplementary Figure 7 Top 30 taxa across four predictive models for 16S studies describing **(A)** general autoimmunity (adults + children). **(B)** Inflammatory bowel disease (adults + children), and metagenomics studies investigating. **(C)** General autoimmunity (adults + children). **(D)** Inflammatory bowel disease (adults + children). **(E)** General autoimmunity (adults only). **(F)** Inflammatory bowel disease (adults only). Features ranked by mean rank across the four models and color indicates the rank of each taxa in each model. Log fold change of disease vs. healthy for each identified taxon is also shown. Features are ranked by mean rank across all four machine learning methods in descending order. ^∗^Indicates that the taxa highlighted was identified as being highly study specific (Supplementary Figure 3).

Supplementary Figure 8 Significant correlations between metagenomic abundance of 6 selected genera and metabolites in IBDMDB dataset.

Supplementary Table 1 Autoimmune diseases samples details including number of studies, data type, number of samples of each data type before and after quality control filtering, and number of samples from adults only (age > 18).

Supplementary Table 2 Details of each of the study including disease type, data type, sequencing platform, instrument, sequencing type, 16S rRNA region, age group, country of samples’ origin, DNA extraction kit, accession numbers, number of the samples before quality control and number of samples used for machine learning.

Supplementary Table 3 Final parameters of predictive models including number of samples, number of features before and after near-zero-variance feature removal and ROC AUC of each model.
