# Supplementary figures and images for: Predictive Metagenomic Analysis of Autoimmune Disease Identifies Robust Autoimmunity and Disease Specific Microbial Signatures
Source: Front Microbiol. 2021 Mar 4;12:621310. doi: 10.3389/fmicb.2021.621310 (PMC7969817; doi:10.3389/fmicb.2021.621310)

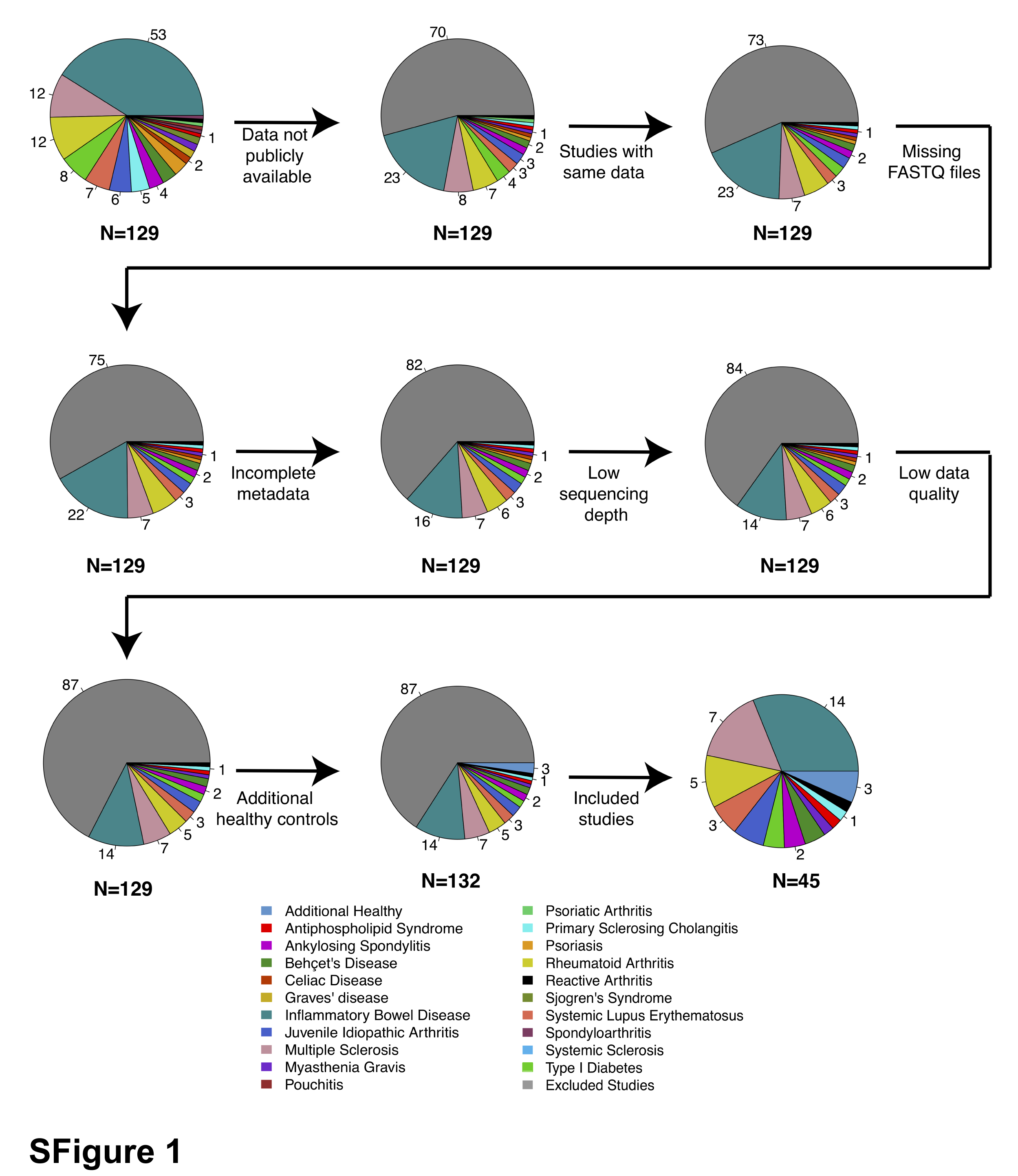

Supplement: Supplementary Figure 1 — Study collection and filtering. [file Image_1.TIF]

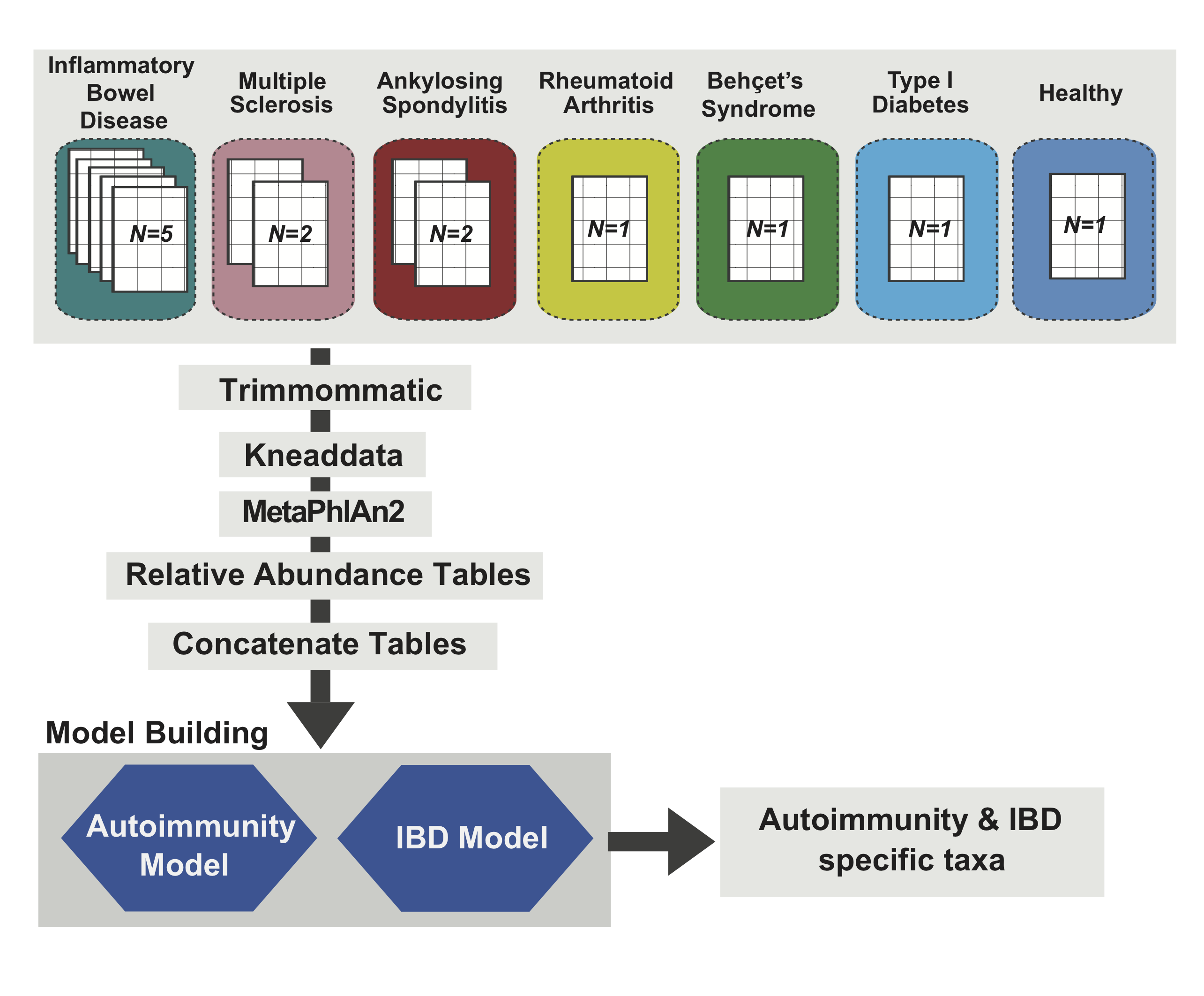

Supplement: Supplementary Figure 2 — Metagenomic analysis workflow. [file Image_2.TIFF]

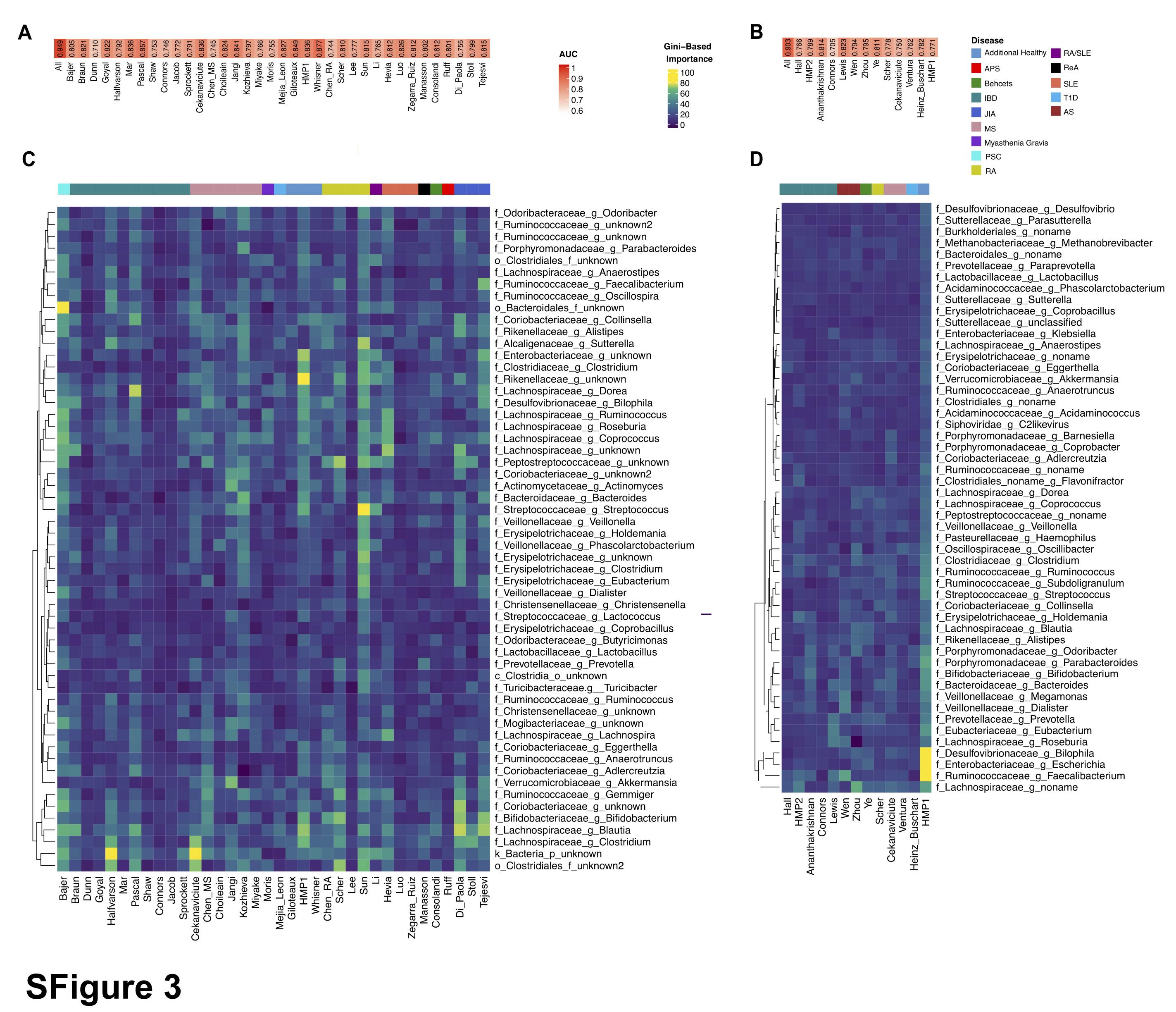

Supplement: Supplementary Figure 3 — Models and features predictive of study. AUCs of random forest model prediction of study and taxa features most predictive of study in those models in 16S (A,C) and metagenomics (B,D) studies. [file Image_3.TIF]

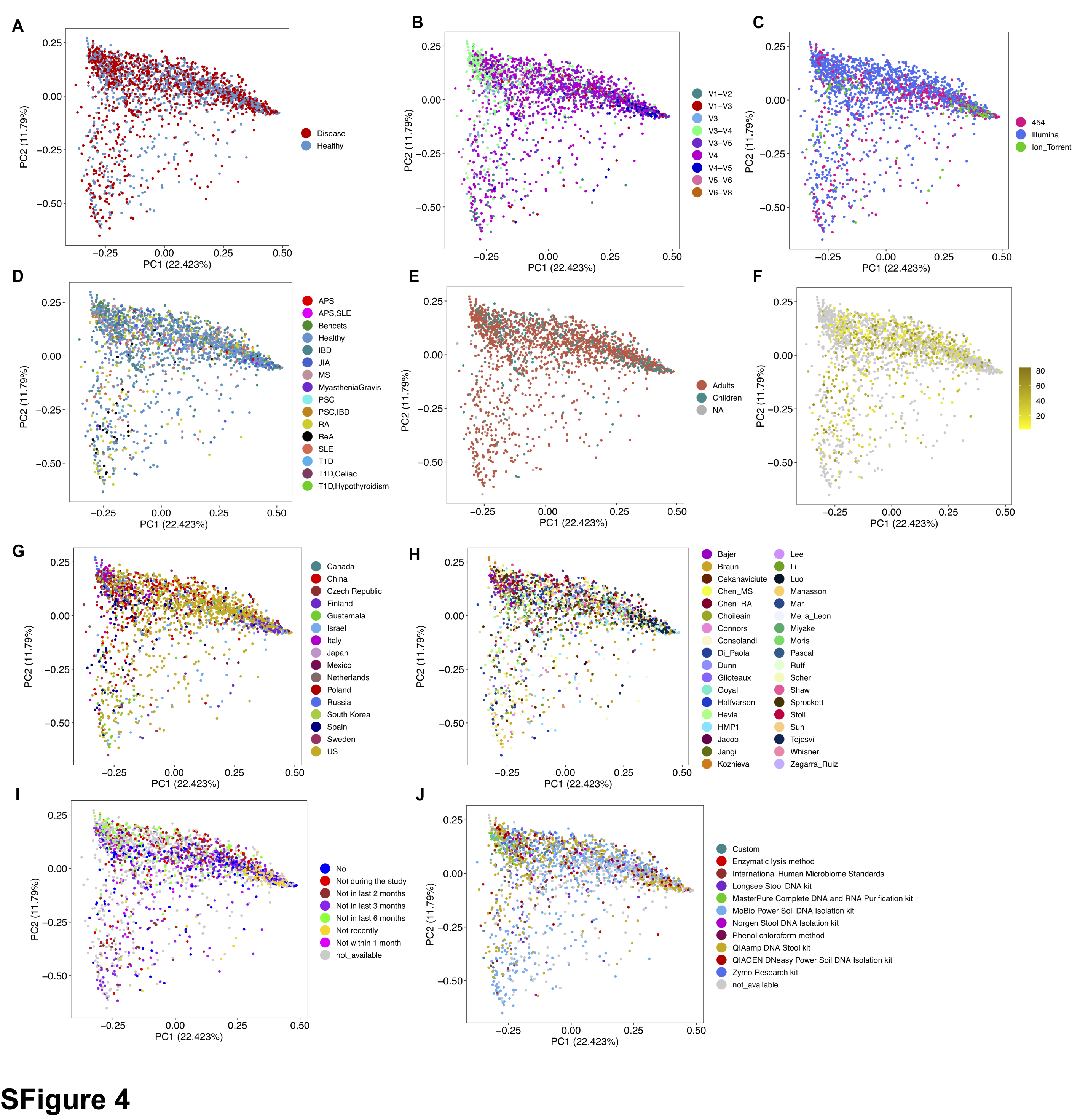

Supplement: Supplementary Figure 4 — PCoA diagrams and statistical differences across 16S datasets showing sample similarity by (A) health status, (B)16S rRNA region sequenced, (C) sequence platform, (D) disease type, (E) age group, (F) exact age, (G) country, (H) study, (I) antibiotics consumption, and (J) DNA extraction kit. All PCoAs have an Adonis p < 0.001. [file Image_4.TIF]

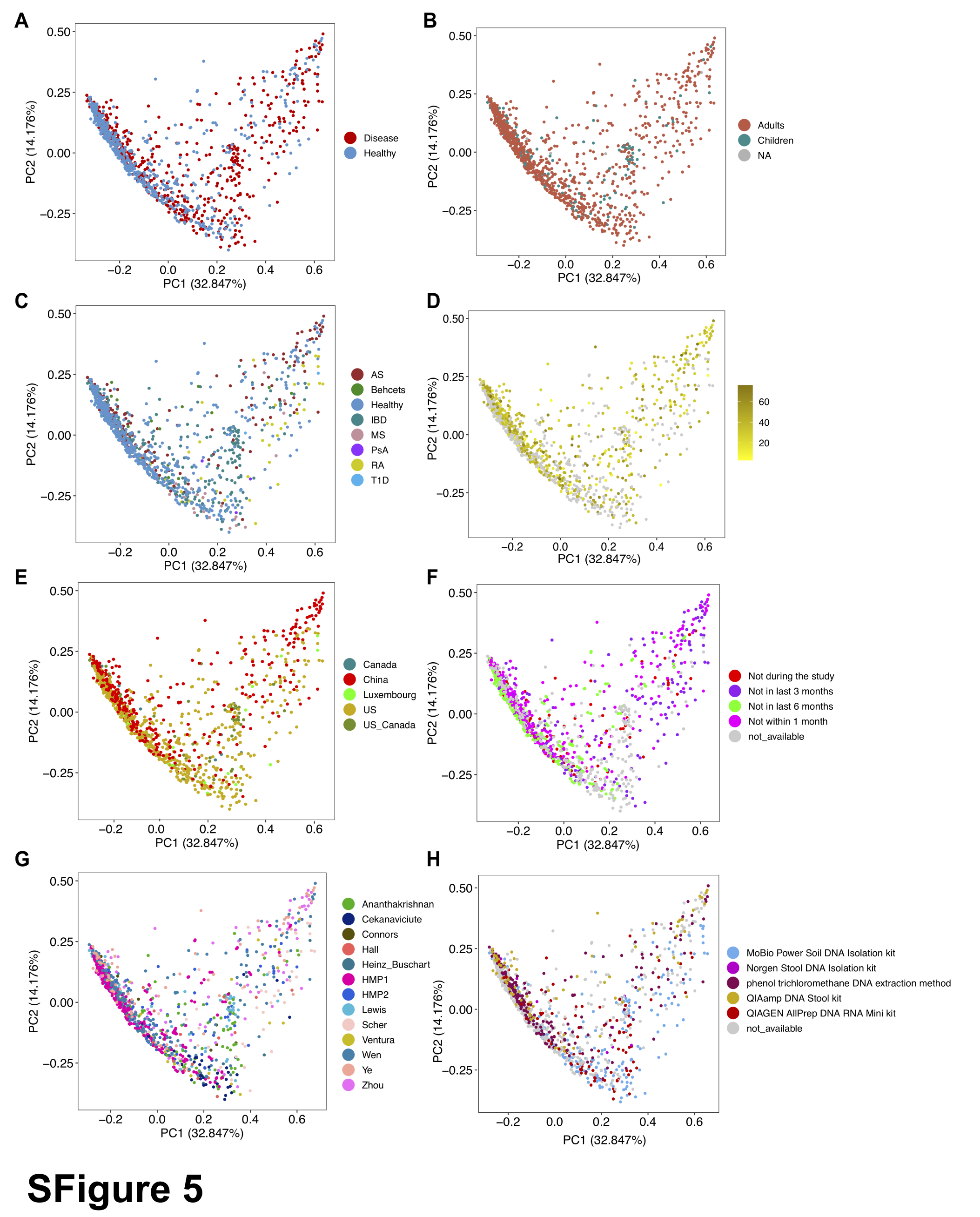

Supplement: Supplementary Figure 5 — PCoA diagrams and statistical differences across metagenomics datasets showing sample similarity by (A) health status, (B) age group, (C) disease type, (D) exact age, (E) country, (F) antibiotics consumption, (G) study, and (H) DNA extraction kit. All PCoAs have an Adonis p < 0.001. [file Image_5.TIF]

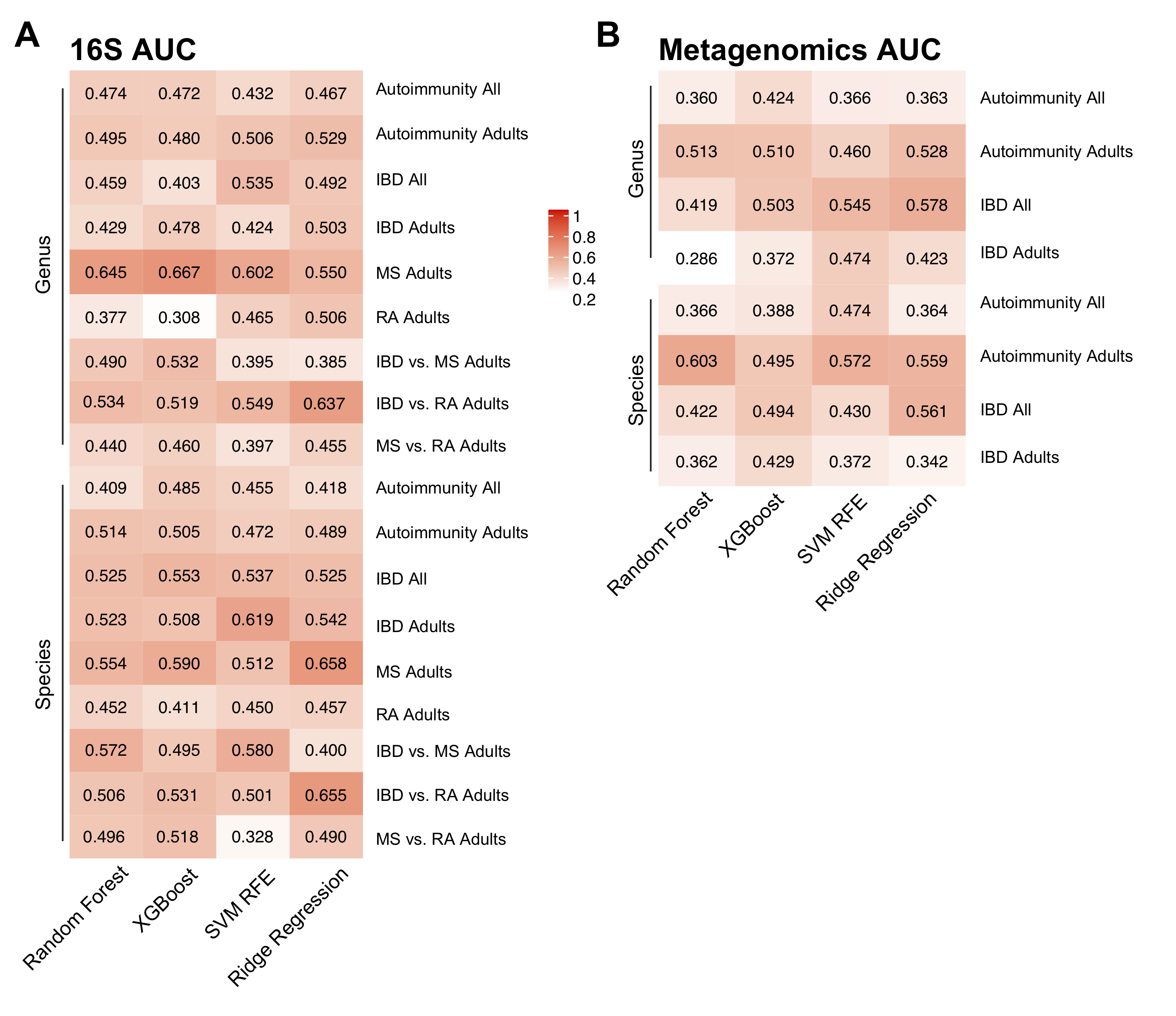

Supplement: Supplementary Figure 6 — AUCs for models trained with random label assignment for (A) 16S and (B) metagenomics studies. [file Image_6.TIFF]

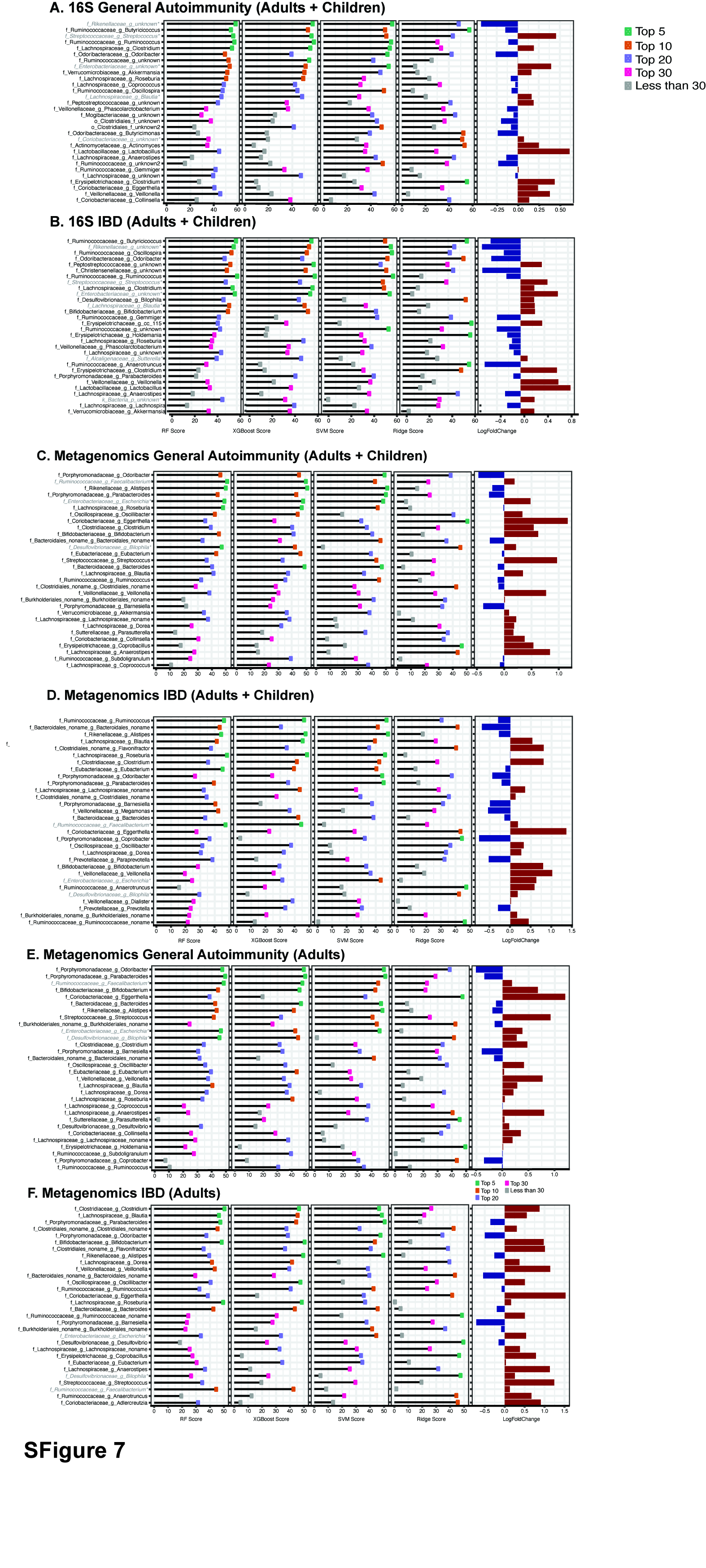

Supplement: Supplementary Figure 7 — Top 30 taxa across four predictive models for 16S studies describing (A) general autoimmunity (adults + children). (B) Inflammatory bowel disease (adults + children), and metagenomics studies investigating. (C) General autoimmunity (adults + children). (D) Inflammatory bowel disease (adults + children). (E) General autoimmunity (adults only). (F) Inflammatory bowel disease (adults only). Features ranked by mean rank across the four models and color indicates the rank of each taxa in each model. Log fold change of disease vs. healthy for each identified taxon is also shown. Features are ranked by mean rank across all four machine learning methods in descending order. ∗Indicates that the taxa highlighted was identified as being highly study specific (Supplementary Figure 3). [file Image_7.TIF]

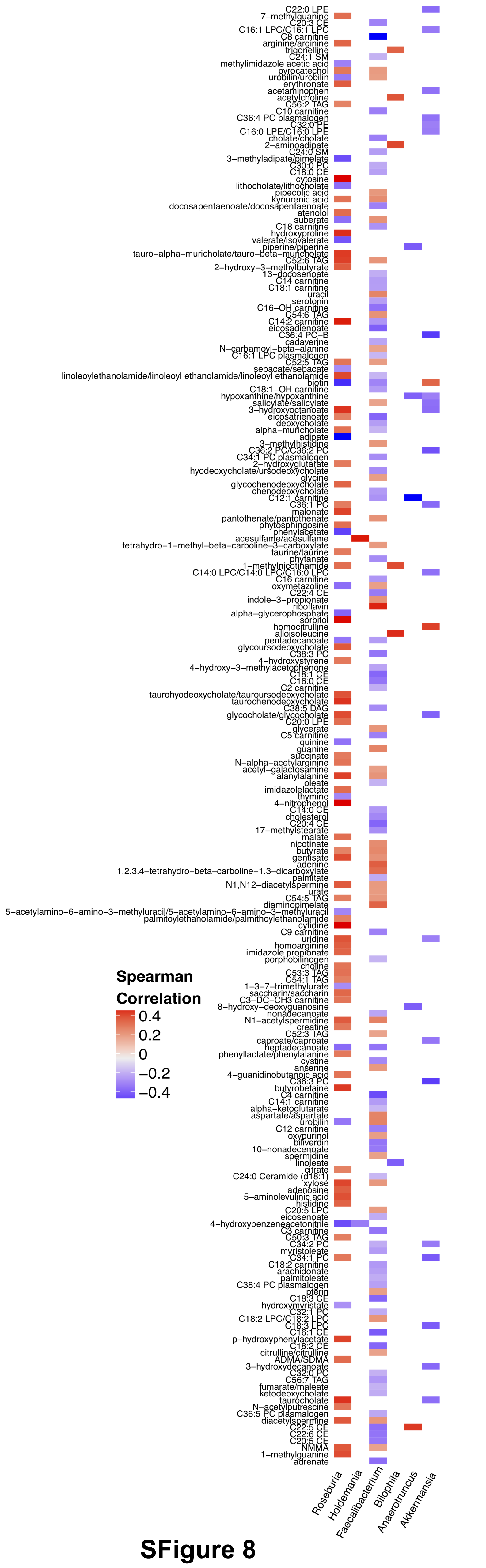

Supplement: Supplementary Figure 8 — Significant correlations between metagenomic abundance of 6 selected genera and metabolites in IBDMDB dataset. [file Image_8.TIF]
